# Development of a Near-Zero-Waste Valorization Concept for Waste NdFeB Magnets: Production of Antimicrobial Fe Alginate Beads via Adsorption and Recovery of High-Purity Rare-Earth Elements

**DOI:** 10.1021/acsomega.3c06178

**Published:** 2024-02-02

**Authors:** Elif Emil-Kaya, Emircan Uysal, Dilara Nur Dikmetas, Funda Karbancioğlu-Güler, Sebahattin Gürmen, Bernd Friedrich

**Affiliations:** † Department of Materials Science and Engineering, Norwegian University of Science and Technology, Trondheim 7491, Norway; ‡ Department of Metallurgical & Materials Engineering, Istanbul Technical University, Istanbul 34469, Turkey; § Department of Food Engineering, Istanbul Technical University, Istanbul 34469, Turkey; ∥ IME Process Metallurgy and Metal Recycling, RWTH Aachen University, Aachen 52062, Germany

## Abstract

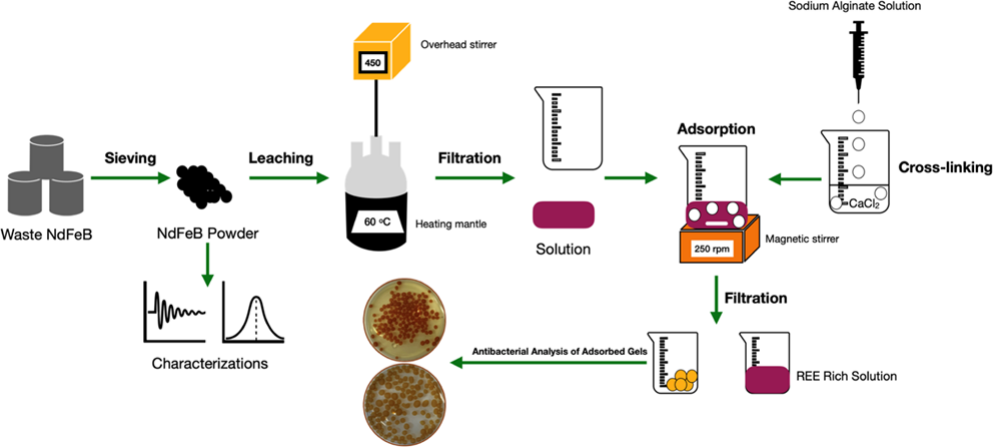

Nowadays, with the evolution of technology, rare earths are raw materials for a multitude of products, especially in high technological applications. A high amount of REEs is used in the production of permanent magnets, particularly NdFeB. The demand for some of the REEs, including neodymium, praseodymium, and dysprosium, is expected to increase in the coming years. REEs are defined as critical materials due to their high supply risk and economic importance. Recycling secondary raw materials for supplying REEs in the future is one promising option, and one of the best candidates is NdFeB magnets. NdFeB magnets include approximately 30% REEs and 66% of iron. For the near**-**zero-waste concept, the recovered iron from NdFeB must be evaluated in other applications. In this study, the near**-**zero-waste valorization concept for EoL-NdFeB magnets is developed, and high-purity REEs are achieved with a two-step process, including leaching and adsorption using alginate beads. Moreover, antimicrobial Fe alginate beads are produced in the leach liquor via adsorption. The antimicrobial activity of the produced Fe alginate beads is evaluated with disc diffusion and broth dilution methods against Gram-positive *Staphylococcus aureus* and Gram-negative *Escherichia coli*. The most effective antibacterial Fe alginate beads are against *E. coli* and *S. aureus* with inhibitions of 87.21 and 56.25%, respectively.

## 1 Introduction

Cobalt, lithium, platinum group metals, tantalum, gallium, and especially REEs are a few important elements that are important to the development of efficient and high-tech applications, such as electric cars that require lithium and neodymium and wind turbines that require neodymium and dysprosium. As the world is shifting toward a “cleaner and greener” future, meeting the growing demand for REEs is becoming increasingly difficult since most of the production is concentrated in a few countries, such as China, the United States, Australia, and India.^1^

That may present a potential problem for meeting global demands since global production hardly covered the demand. China extracted and processed a large amount of rare earth oxides (REOs), which greatly surpasses the rest of the world’s supply, and then exported a quantity after meeting their demands. Hence, in many studies, Nd, Pr, Dy, Eu, and Tm are considered critical metals because of supply risks and economic importance, which reflects the vulnerability of the economy to potential shortage or supply interruption creating a surge in prices.^2−4^ A high amount of REEs is used in permanent magnet production, including NdFeB and SmCo. Therefore, recycling of NdFeB is one of the best options for the supply of REEs in the future.

In the literature, many researchers investigate the recovery of REEs by hydrometallurgical methods, including leaching, solvent extraction, precipitation, ionic liquids, etc.^5,6^ These methods have several drawbacks. For instance, the precipitation process necessitates the utilization of several chemicals and the resultant precipitant lacks adequate purity. Furthermore, methods capable of obtaining high purity, such as solvent extraction, possess certain drawbacks due to the requirement of a large number of process steps and the necessity of complex chemicals. Due to the disadvantages of these methods, new methods must be developed.

The adsorption method has recently emerged as a promising method in solution metal enrichment processes.^7^ During the process of adsorption, a layer of metal ions, referred to as adsorbate, is deposited onto the surface of adsorbents.^8^ Strong cation exchange resins,^9^ layered double hydroxides,^10^ and low-cost biosorbents like alginate^11^ are a few particular adsorbents that have been investigated for metal ion separation. Although adsorption is a commonly used technique for removing metal ions from wastewater due to its simplicity, convenience, and effectiveness,^12^ only a few research based on adsorption have been carried out with no recovery of REEs from waste NdFeB magnets. Ion-imprinted mesoporous silica was reported as an adsorbent for the adsorption of dysprosium from the acidic NdFeB magnet leachate.^13^ In another study, silicas with phosphonate and amino polycarboxylate ligands were employed for the recovery of REEs from leach solutions of real NdFeB.^14^ Modified mesoporous silica was used in these studies, but from our perspective, no previous work has been done with a biocompatible and biodegradable material for waste NdFeB magnets.

Alginate is a biocompatible and biodegradable polysaccharide used in adsorption processes and consists of 1,4-d-mannuronic (M) and -l-guluronic (G) acid groups.^15^ Alginate can be found in nature in the form of sodium alginate.^15^ Sodium alginate is the form of carboxyl groups in the alginate chain structure in the ionic interaction with sodium. Sodium alginate is water-soluble^16^ and unstable in the aqueous environment. The stability of alginate in water must be increased in order to perform adsorption studies. Due to the existing carboxyl groups, alginate can become rigid and forms a hydrogel by interacting with divalent or trivalent metal ions instead of sodium ions, creating a structure called “Egg-Box”.^17^ Alginate has been widely researched for its capacity to absorb heavy metal ions. Due to the high capacity of alginate to bind divalent and trivalent metal ions, the adsorption of Cu, Pb, Cd, and Hg ions in solution has been studied.^11^ It is also known that the obtained metal-alginate gels show antibacterial properties.^16^ Uysal et al. reported that Cu alginate and Fe alginate gels showed antibacterial properties for Cu against *Escherichia coli* and *Staphylococcus aureus* and Fe against *S. aureus*.^16^

Herein, a novel near-zero-waste valorization concept for waste NdFeB magnets is proposed for the recovery of high-purity REEs. First, the magnet powders are leached by nitric acid, and in this process, some amounts of iron are separated as leach residue through in situ iron hydrolysis during leaching. The leach residue is calcined to obtain Fe_2_O_3_ that can be used as a pigment. Afterward, the remaining iron in the leach solution is adsorbed by using CaAlg beads. Compared to conventional metal enrichment methods, REE enrichment is achieved through adsorption, which proves to be a cheaper, more convenient, and environmentally friendly method. High-purity REEs are achieved, and antimicrobial Fe alginate beads are fabricated through adsorption. The antimicrobial efficiency of the Fe alginate beads is studied with disc diffusion and broth dilution methods against Gram-positive *S. aureus* and Gram-negative *E. coli*. A pilot-scale setup for producing antimicrobial Fe alginate beads is proposed.

## 2 Materials and Methods

### 2.1 Magnet Powder Preparation and Characterization

Waste NdFeB magnets were supplied in bulk form, as rectangles, from various companies. The supplied waste magnets were crushed by a jaw crusher (Retsch BB51) and sieved by a vibratory sieve shaker (Retsch, AS200) to acquire a fine powder. Nitric acid (65%) from Sigma-Aldrich was used for leaching experiments. The chemical composition of the magnet powders was analyzed by inductively coupled plasma optical emission spectroscopy (ICP-OES, Spectro Arcos Analytical Instruments). For ICP-OES analysis, approximately 6.69 g of the magnet powder was dissolved in 200 mL of 2 mol/L nitric acid solution for a 1/30 solid-to-liquid ratio for 30 min, and the magnet powders were allowed to dissolve almost completely into the solution. Morphological investigation of NdFeB and elemental mapping of these powders were conducted by field-emission scanning electron microscopy (Thermo Fisher Quattro S, FESEM).

### 2.2 Leaching of NdFeB Magnet Powders with Nitric Acid

The leaching study was performed as optimized in our previous study.^18^ Briefly, the magnet powders were leached in a 1.82 mol/L acid concentration with a solid-to-liquid ratio of 11:100 and a stirring speed of 415 rpm at a process temperature of 60 °C. A heat-controlled mantle was utilized with a three-neck quartz reactor with constant overhead stirring at 450 rpm. In addition, a condenser with a constant water throughput was employed. First, the water quantity was introduced to the reactor, followed by the acid, and stirred for 1 min to mix them. Then, the powder was introduced, stirred for a few seconds, and allowed to cool since mineral acid leaching is an exothermic process. Afterward, solid and liquid separation was performed by a vacuum pump.

### 2.3 Production of CaAlg Beads

Sodium alginate with a low viscosity was kindly provided by Alfa Aesar. Sodium alginate was dispersed at a speed of 350 rpm with a magnetic stirrer in distilled water at a concentration of 1 g/100 mL at 90 °C. The solution was left to stand for approximately 3 h to remove bubbles. A syringe pump was employed to produce sodium alginate beads. A calcium chloride dihydrate solution at a concentration of 15 g/L was prepared by dissolving its powder in distilled water. The alginate gel was dropped into a calcium chloride dihydrate solution. The reaction between Ca and alginate proceeded and the formed beads were left in a calcium chloride dihydrate solution for 24 h.

### 2.4 Adsorption Studies of Iron from the Leaching Solutions

The adsorption studies were performed in a series of 50 mL beakers containing various amounts of Ca alginate beads. The effect of time, solid-to-liquid ratio, temperature, and concentration of the solution on the adsorption of iron was investigated in detail. Table 1 presents the experimental parameters for the adsorption studies.

**Table 1:** Experimental Parameters of the Adsorption Studies

experiment code	S/L ratio (g/mL)	temperature (°C)
A1	1/10	20
A2	1/20	20
A3	1/30	20
A4	1/10	40

The metal ion concentration in the solution obtained after the leaching process is given in Table 3. The solution used in the adsorption experiments was prepared by diluting the leaching solution 4-fold. Each adsorption experiment occurred with a 20 mL prepared solution, a magnetic heating stirrer (model: MS300HS-MTOPS), and a stirring speed of 250 rpm. To determine the time, the first adsorption experiment was carried out for 180 min (20 mL solution, 20 °C, and 1/10 solid-to-liquid ratio). As will be mentioned in the following sections, since the maximum efficiency is realized in the 120th minute, the time of the adsorption process was determined as 120 min in all further experiments. Furthermore, to examine the effect of the solid-to-liquid ratio, three different solid/liquid ratios were used (1/10, 1/20, and 1/30). Equation 1 is used to calculate the adsorption efficiency^19^
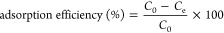
1

where *C*_0_ is the concentration of metal ions in the solution (mg/L) and *C*_e_ is the metal ion concentration of the solution (mg/L) in the equilibrium system. The adsorption capacity of adsorbent material is calculated using eq 2(19)
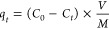
2where *q_t_* is the adsorption capacity of the adsorbent material at a specific time (mg/g), *V* is the volume of the solution (L), *M* is the quantity of the adsorbent (g), *C*_0_ is the starting concentration of the solution (mg/L), and *C_t_* is the concentration of the solution at a specific time (mg/L). The volumetric coefficients in eqs 1 and 2 were calculated by considering the factors that influence the volume change, including sampling.

#### 2.4.1 Adsorption Kinetics

The first experiment, which was conducted using 0.2 g of adsorbent and a 20 mL solution at a temperature of 20 °C, provided for the kinetics of the adsorption study to be determined by taking samples from the solution at various time intervals. The linearized pseudo-first-order equation, linearized pseudo-second-order equation, nonlinear pseudo-first-order equation, and nonlinear pseudo-second-order equation are the four theorems that are used most to explain adsorption kinetics. These 4 different methods can be used to express adsorption kinetics. Separate analyses were conducted to determine whether the 4 different models are suitable for the experimental data. With the nonlinear pseudo-first-order equation, the adsorption kinetics were fitted with eq 3(20)
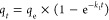
3

In this equation, the terms *q*_e_ and *q_t_* refer to the equilibrium and “*t*” adsorption capacities, respectively (mg/L). *k*_1_ is the equation’s constant, and *t* is the duration in minutes. The rate of change of ion adsorption with time is given by the pseudo-first-order equation. The linearized pseudo-first-order represents via eq 4(20)
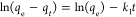
4

The variables are the same as the nonlinear equation, also *k*_1_ and *q*_e_ values were calculated by plotting ln(*q*_e_ – *q_t_*) versus time. Nonlinear pseudo-second-order is fitted with experimental data via eq 5(20)
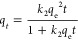
5where variables are the same as pseudo-first-order. Equation 6 represents linearized pseudo-second-order^20^
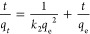
6

Adsorption reactions fitted to the pseudo-second-order are chemisorption-controlled.

#### 2.4.2 Adsorption Isotherms

Adsorption isotherms provide the basis for explaining how adsorbent and pollutant materials interact. It investigated how isotherm models and experimental data related. The Langmuir and Freundlich isotherm models were found to have a higher agreement with the experimental results in this investigation. The agreement between experimental results and the Langmuir isotherm is examined in eq 7(21)
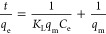
7

here, *q*_m_ is the maximum adsorbent capacity of the adsorbent (mg/g), *C*_e_ is the equilibrium concentration (mg/L), and *K*_L_ is the Langmuir constant. It is possible to think that homogeneous adsorption takes place on the adsorbent surface and in the monolayer for adsorption investigations in line with the Langmuir model. Furthermore, eq 8 shows the Langmuir separation factor
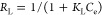
8

The *R*_L_ number describes the kind of adsorption, which can be either irreversible (if *R*_L_ = 0), linear (if *R*_L_ = 1), unfavorable (if *R*_L_ > 1), or favorable (if *R*_L_ = 1).^22^ Additionally, eq 9 can be used to investigate the correlation between the Freundlich isotherm and experimental data^21^
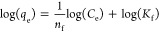
9

In this case, the Freundlich constant *K*_f_ and the Freundlich isotherm constant *n*_f_ provide information regarding the feasibility of adsorption, in contrast to the Langmuir isotherm. Additionally, if 1/*n*_f_ is less than 1, typical adsorption is predicted; however, if *n*_f_ is equal to 1, the partition between the two phases is concentration-independent.^22^ To understand both isotherm models, experiments were carried out with solutions with three different dilution ratios of 20 mL (4, 2, and 1 times) at a 1/10 solid-to-liquid ratio for 120 min. The Langmuir model was fitted by plotting 1/*q*_e_ versus *C*_e_, and Freundlich by plotting ln *q*_e_ versus ln *C*_e_; also *K*_L_ and *K*_f_ constants were calculated.

### 2.5 Effect of Temperature on Adsorption Temperature on Adsorption Reaction and Thermodynamic Constant

Equaions 10 and 11 estimated the Gibbs free energy change (Δ*G*°) of adsorption studies; eq 12 calculated Δ*H*° and Δ*S*°^23^
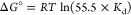
10
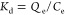
11
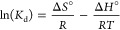
12

The value of *K*_d_ in the equation denotes the adsorption equilibrium constant. By finding Δ*G*°, it is possible to determine whether the system arises spontaneously or not.

### 2.6 Characterization of Ca/Fe Alginate Beads

The thermal decomposition behavior of the produced CaAlg and FeAlg beads was investigated by differential thermal analysis and thermogravimetric analysis (DTA-TG; Netzsch STA 409, Selb, Germany) using an alumina crucible. These measurements were performed under an Ar atmosphere with a flow rate of 50 mL/min. Heating was performed from 25 to 800 °C with a heating rate of 10 °C/min. FTIR spectra of CaAlg and FeAlg beads were recorded by Fourier-transform infrared (FTIR) spectroscopy (Bruker α-T).

### 2.7 Evaluation of the Antimicrobial Activity of Fe Alginate Beads

The antibacterial activities of the iron-adsorbed materials were tested against Gram-positive *S. aureus* ATCC 25923 and *E. coli* ATCC 25922^16^ with a disc diffusion method using Luria–Bertani Agar. *E. coli* and *S. aureus* were grown overnight in LB broth at 37 °C for 24 h. After incubation, bacterial suspension concentrations were adjusted to 1 × 10^5^ CFU/mL using sterile saline (0.85% NaCl) water. Afterward, 100 μL of bacterial suspension was spread on LB agar plates. The samples were sterilized using a UV cabinet, placed onto the LB agar plates, and incubated at 37 °C for 24 h. After incubation, the inhibition zone diameter for each material was measured and recorded. The analysis was conducted in four parallels.

The broth microdilution method was used to determine the inhibitory percentages of the material with slight modifications by Arafa et al.^24^ Bacterial strains were grown on Mueller Hinton broth at 37 °C for 24 h. The sample concentration was prepared with a 10% DMSO solution at 125 μg/mL and the 10% DMSO solution was used as the negative control. 180 μL of the samples was added to each well in a 96-well plate, and then 20 μL of the prepared bacterial solution was added to the plate. Positive control of the samples contains a bacterial suspension without the material. After incubation at 37 °C for 24 h, the absorbance was measured at 600 nm using a UV–vis spectrophotometer (Synergy HT, BioTek Instruments Inc.). The inhibition percentage of the samples was calculated according to the following eq 13
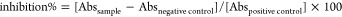
13Abs_sample_ indicates that wells contain sample and microorganism, Abs_negative control_ indicates that wells contain sample alone, and Abs_positive control_ that wells contain microorganism.

### 2.8 Statistical Analysis for Antimicrobial Analysis

A statistical analysis method (one-way ANOVA) was used to assess the significance of the antimicrobial analysis with Tukey’s test using SPSS 22.0 (SPSS Inc., Chicago, IL). *p* < 0.05 was indicated as the level of significance.

## 3 Results and Discussion

### 3.1 Characterization of NdFeB Magnets

NdFeB magnets were crushed to obtain powder by a jaw crusher (Retsch BB51). After the magnet powders were crushed, the powders were sieved by a vibratory sieve shaker (Retsch, AS200). The particle size of the sieved magnet powders was smaller than 90 μm. Afterward, XRF analysis was performed to determine the chemical composition. Table 2 shows the XRF analysis of the magnet powders.

**Table 2:** Chemical Composition of NdFeB Magnet Powder Was Determined by XRF Analysis

component	Na_2_O	Al_2_O_3_	SiO_2_	MnO	Fe_2_O_3_
concentration (%)	0.34	0.42	0.24	1.97	68.1

component	Co_3_O_4_	CuO	Ga_2_O_3_	As_2_O_3_	Nb_2_O_5_
concentration (%)	0.70	0.14	0.20	0.21	0.12

component	PdO	Pr_2_O_3_	Nd_2_O_3_	Tb_4_O_7_	other
concentration (%)	0.24	5.72	20.4	0.70	0.50

As reported elsewhere,^5,6^ high amounts of Fe, Nd, and Pr are observed, with the addition of small amounts of Dy, Si, Al, Co, Mn, and Pd.

**Figure 1:**
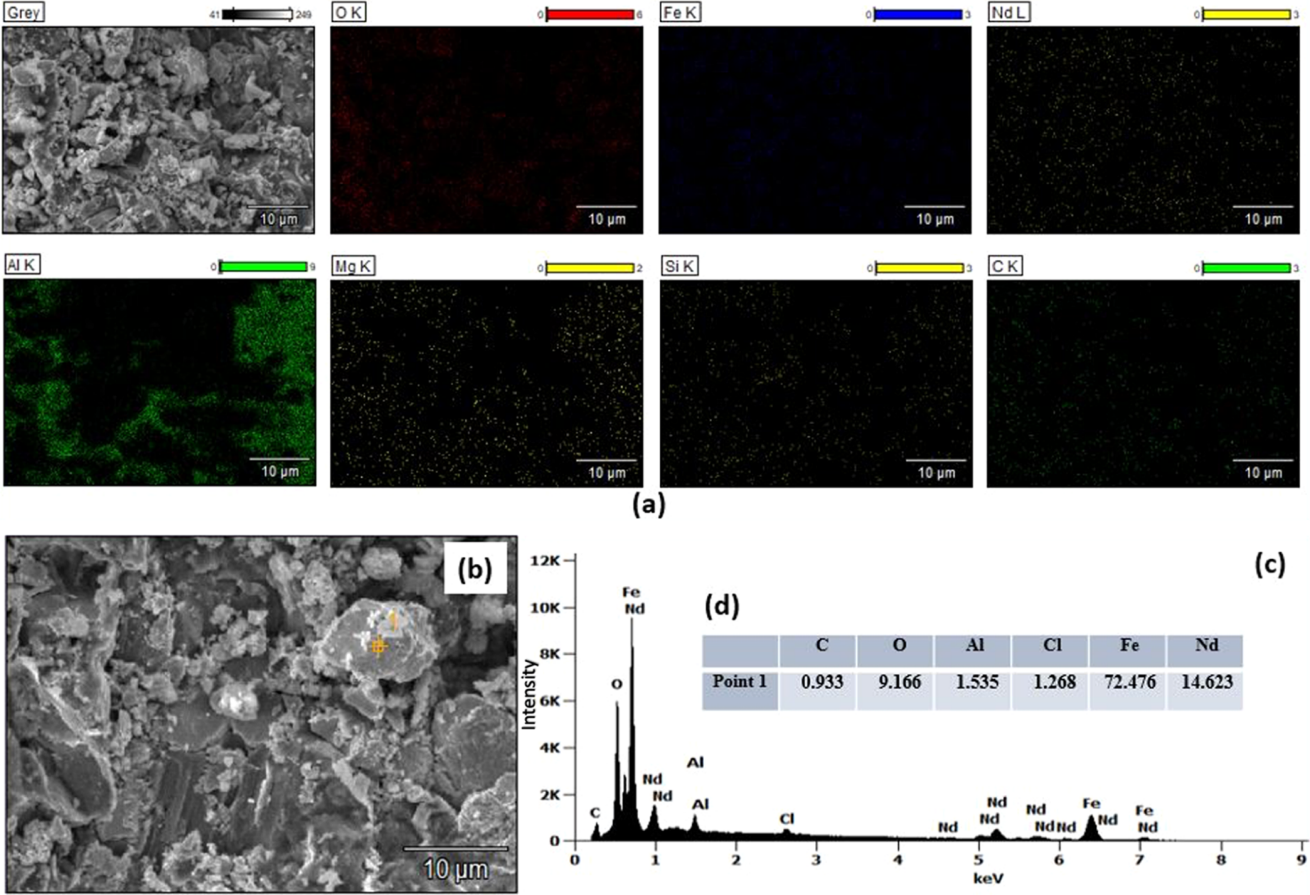
(a) Elemental mapping of the magnet powders, (b) FESEM micrograph of the magnet powders, (c) EDX point analysis of the magnet powders (percentage weight), and (d) EDX analysis of the magnet powders.

The morphology of the magnet powders was revealed by field-emission scanning electron microscopy (Thermo Fisher Quattro S, FESEM). Elemental mapping of the NdFeB magnet powder was conducted by EDX (energy-dispersive X-ray) analysis. Figure 1 illustrates elemental mapping of the magnet powders (a), FESEM micrographs of the magnet powders (b), EDX point analysis of the magnet powders (percentage weight) (c), and EDX analysis of the magnet powders (d).

O, Fe, Nd, Si, and Mg were detected by EDX analysis. Al–Mg alloy tub was used for SEM and EDX analyses; therefore, small amounts of Al and Mg were observed, as well. For the detailed chemical analysis of the magnet powders, ICP analysis was carried out as well. Table 3 represents the result of the ICP-OES analysis of NdFeB magnet’ powders. It was determined that Fe was the primary element, with REEs identified as secondary elements in all three analytical methods. The differences among all three elemental analyses arise from the different working principles of the analysis methods. The map detailing the distribution of elements in the material was acquired through EDX analysis. The XRF analysis provided general information about the element distribution in the material. The distribution of elements present in the material was determined by taking accurate measurements at the parts per million level, utilizing ICP analysis. Since ICP analysis enables more precise measurements, the study continued with the elemental composition determination of the magnets using this method.

**Table 3:** Chemical Analysis of NdFeB Magnet Powder Was Determined by ICP Analysis

composition	B	Co	Cr	Cu	Dy
concentration (%)	0.877	0.773	<0.1	0.102	0.662

composition	Fe	Mo	Nd	Ni	Pr
concentration (%)	66.27	<0.1	23.9	<0.1	7.38

### 3.2 Leaching of NdFeB Magnet Powders

In this study, an oxidative leaching strategy was proposed to promote in situ iron hydrolysis during leaching. Minimization of iron in the system was achieved through hydrolysis during oxidative leaching.^18^ During the oxidative leaching process, NO*_x_* gases were released, and the gases were captured with the washing bottles to produce nitric acid, thereby achieving a closed loop for nitric acid. Table 4 shows the chemical composition of the leaching solution.

**Table 4:** Chemical Composition of the Leaching Solution

	Nd	Pr	Fe	Dy	Co	B
leaching solution	1.80 g/L	0.56 g/L	0.67 g/L	69.7 mg/L	53.3 mg/L	51.0 mg/L

A high amount of Nd, Pr, and Fe was observed and a small amount of Dy, Co, and B was detected by ICP analysis.

### 3.3 Adsorption of Fe^2+^/Fe^3+^ and REE^3+^ Ions from the Leaching Solution

#### 3.3.1 Adsorption Time

**Figure 2:**
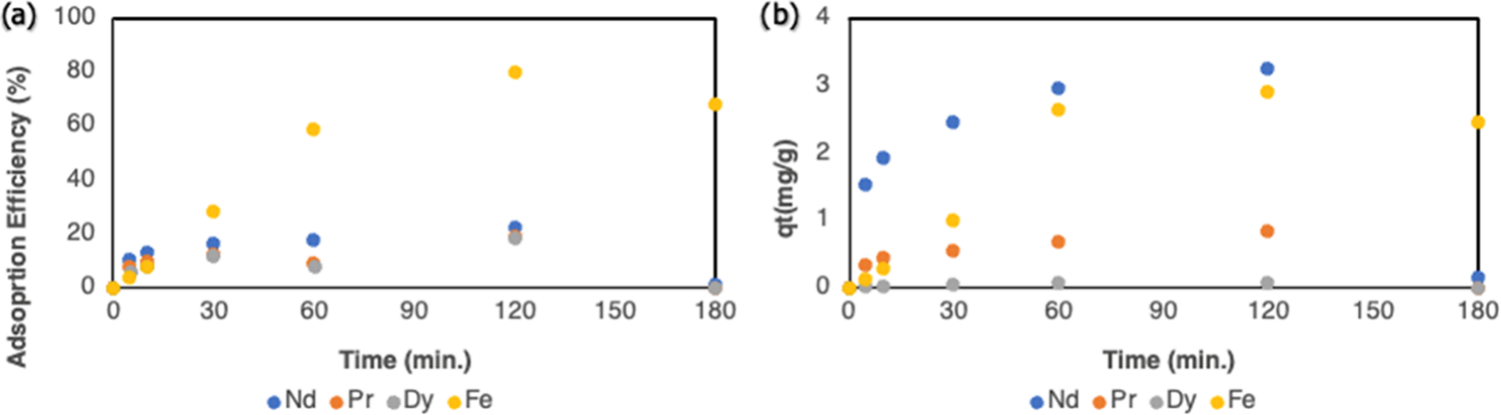
Time-dependent adsorption efficiencies (a) and adsorbent capacity of gels for each metal ion (b).

Since the contact time affects the adsorption process, it should be optimized. To optimize the adsorption time, the adsorption process was carried out for 3 h with a 1/10 solid-to-liquid ratio and a 20 mL solution at 20 °C. The adsorption capacity of the adsorbent CaAlg beads and the change in adsorption efficiency over time are given in Figure 2. It was observed that the adsorption efficiency and absorbent capacity reached the maximum value at the 120th minute and decreased dramatically by two values at the 180th minute. Carboxylate groups in the alginate structure are protonated at low pH,^17^ thus damaging the calcium alginate backbone structure; also, low pH causes shrinkage of calcium alginate and changes the morphology of calcium alginate beads.^25^ To prevent the decrease in the efficiency due to the calcium alginate structure starting to deteriorate after the 120th minute and the adsorption efficiency reaching its maximum value in the 120th minute, the time was chosen as 120 min in further experiments. The adsorption efficiencies for Nd, Pr, Dy, and Fe ions, at the 120th minute, were observed as 22.44, 19.65, 18.45, and 80.54%, respectively. In addition, the adsorbent capacity was observed as 3.26, 0,86, 0.10, and 2.91 mg/g for Nd, Pr, Dy, and Fe, respectively. The adsorbent capacity, which is the quantity of adsorbate absorbed by the adsorbent per unit mass or volume of the adsorbent, can be influenced by several factors. pH, dosage, agitation speed, time, temperature, particle size, surface area, and micropore volume of the adsorbent are the parameters that affect the absorbent capacity.^26−28^ In addition, different properties of different metal ions, such as their ionic radius, charge, and electronegativity, change the adsorbent capacity of each metal ion in the same material.^29,30^ Therefore, the adsorption capacity for each metal ion is different and can be listed as Nd > Fe > Pr > Dy in these conditions.

#### 3.3.2 Adsorption Kinetics

**Figure 3:**
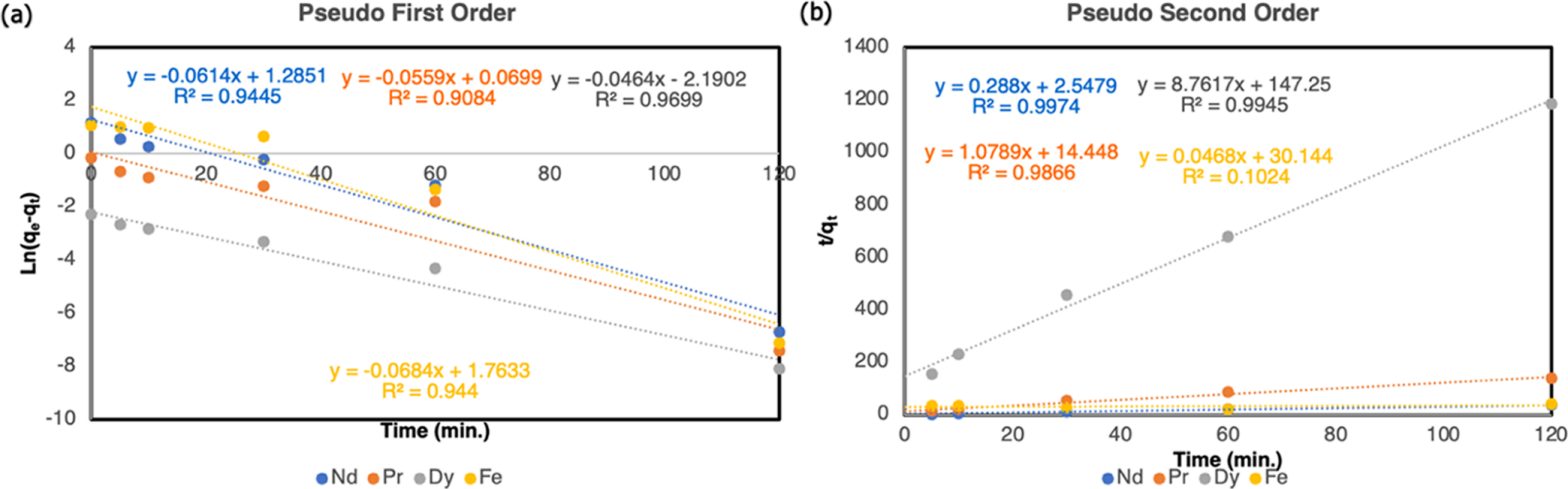
Compatibility of experimental data with different kinetic models: Pseudo first order (a) and pseudo second order (b).

To better understand the absorption behavior, linearized pseudo-first-order and linearized pseudo-second-order kinetic models were applied for the adsorption process of each metal ion (see Figure 3). The constants of two different kinetic models and *R*^2^ values for all metal ions were calculated and are given in Table 5. The *R*^2^ values for the adsorption kinetics of REE ions were compatible with the pseudo-second-order, and the effective adsorption mechanism was chemisorption. However, it was understood that for the Fe ions’ adsorption process, the pseudo-first-order was compatible with experimental data. It was observed that the adsorption of Fe ions depends on the solution concentration. The reason why Fe and REE ions fit different kinetic patterns is that while REEs have similar chemical and physical properties, Fe ions exhibit different chemical and physical properties. The “3+” valence ionic radii of REE metals are close to each other and are larger than the radius of Fe “2+ and 3+” ions. In the study by Hamed et al., using hydrogel-based materials with magnetic properties, they investigated the coadsorption of Ce^3+^ and Fe^3+^ ions, and the yield was calculated as 80.1% for Ce^3+^ ions and 19.1% for Fe^3+^ ions (at pH = 4).^19^ In this study, in which the magnetic adsorption method was used, the kinetic models for both metal ions were determined as the pseudo-second-order. Unlike this study, in which chemisorption-controlled adsorption was performed, Fe ions were adsorbed in a concentration-controlled manner in our study. This explains why the amount of Fe ions is high in addition to the adsorbed REEs in our study. Furthermore, while the *q*_e_ values calculated with the pseudo-second-order of REE^3+^ ions and the experimental *q*_e_ values were close to each other, a large difference was observed between the *q*_e_ values calculated with the pseudo-first-order and the experimental *q*_e_ values of Fe^2+/3+^ ions. The reason is that before the system reaches equilibrium, the alginate structure deteriorates, and the adsorption process is adversely affected in a way that the adsorption kinetic model cannot predict.

**Table 5:** Calculated Constants of Kinetic Model Equations

	pseudo first order			pseudo second order		
model	*q*_e_ calculated (mg/g)	*k*_1_ (min^–1^)	*R*^2^	*q*_e_ calculated (mg/g)	*k*_2_ (g/mg·min)	*R*^2^
Nd	3.6002	0.0614	0.9445	3.4722	0.0325	0.9974
Pr	1.0724	0.0559	0.9084	0.9226	0.0813	0.9866
Dy	0.1189	0.0464	0.9699	0.1141	0.5216	0.9945
Fe	5.8916	0.0684	0.9440	21.36	0.0001	0.1024

#### 3.3.3 Effect of Solid-to-Liquid Ratio on Adsorption

To examine the adsorption effect of the solid-to-liquid ratio, two more solid–liquid ratios were tried (1/20 and 1/30), different from 1/10 at 20 °C, with 20 mL solutions with the same concentration. The adsorption efficiencies for 3 different solid–liquid ratios are given in Table 6. Since the adsorption sites increased with the increasing adsorbent material, reducing the solid-to-liquid ratio to values less than 1/10 decreased the adsorption efficiency.

**Table 6:** Adsorption Efficiencies for Different Solid-to-Liquid Ratios (20 mL Solution, Same Concentration, and 20 °C)

	adsorption efficiency (%)			
solid/liquid ratio	Nd	Pr	Dy	Fe
1/10	25.89	23.74	22.26	76.17
1/20	4.88	4.88	5.12	54.37
1/30	0.00	0.00	0.00	29.93

#### 3.3.4 Effect of Initial Metal Concentration on Adsorption Capacity

**Figure 4:**
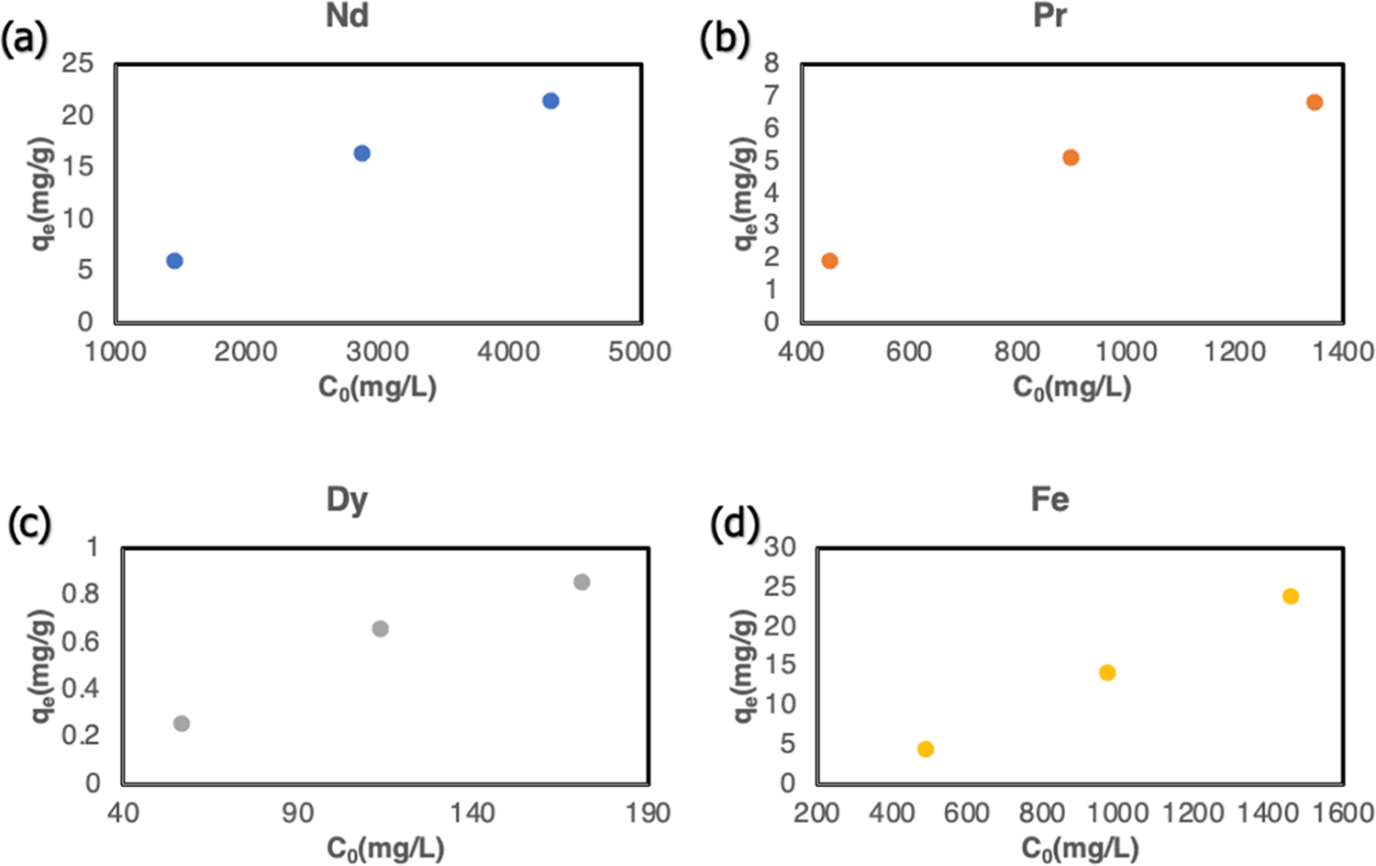
Variation of adsorption capacity with the initial concentration: Nd (a), Pr (b), Dy (c), and Fe (d) (120 min, 20 mL solution, 2 g of adsorbent, 20 °C).

A solution with 3 different concentrations was prepared with the starting solution, and 3 different adsorption experiments were carried out for 120 min at 20 °C and with a 1/10 solid-to-liquid ratio. The variation of *q*_e_ for 4 different metal ions concerning *C*_0_ is given in Figure 4. The adsorbent capacity increased with increasing initial metal ion concentration due to the increased adsorbent and metal ion interaction.^19^

#### 3.3.5 Effect of Temperature and Thermodynamic Constants

The effect of maintaining constant adsorption parameters while increasing the adsorption temperature to 40 °C on the adsorption efficiency of metal ions and the calculated values of Δ*G*°, Δ*H*°, and Δ*S*° for the metal ions are presented in Table 7. It was observed that increasing the temperature decreased the REE^3+^ adsorption efficiency and increased the Fe^2+/3+^ adsorption efficiency. Furthermore, it was observed that all metal ions exhibited negative Gibbs free energy values at both 20 and 40 °C. However, upon examination of their enthalpies, it was found that REE ions displayed an exothermic behavior, whereas Fe ions exhibited an endothermic behavior. Moreover, it was observed that the adsorption of Fe ions occurred in the direction of entropy, whereas REE ions did not exhibit such behavior.

**Table 7:** Effect of Temperature on Adsorption Efficiencies (20 mL Solution, Same Concentration, and a 1/10 Solid-to-Liquid Ratio) and Thermodynamic Constant for Adsorption Reaction

	Nd	Pr	Dy	Fe
Adsorption Efficiency (%)				
20 °C	25.89	23.74	22.26	76.17
40 °C	6.80	2.46	0.51	86.41
Δ*G*° (kJ/mol)				
20 °C	–14.23	–14.64	–14.83	–7.75
40 °C	–18.79	–21.55	–25.69	–7.17
Δ*H*° (kJ/mol)	–52.49	–86.61	–144.28	16.37
Δ*S*° (kJ/mol·K)	–0.23	–0.35	–0.54	0.03

The optimum experimental parameters for Fe adsorption were determined to be a solid/liquid ratio of 1/10 at 40 °C. Under these conditions, more than 86% Fe was removed from the system. However, a two-step adsorption process is proposed to remove all iron. For the second step, the optimum parameters for the removal of the remaining Fe are determined to be a solid/liquid ratio of 1/30 at room temperature.

#### 3.3.6 Determination of Adsorption Isotherm

**Figure 5:**
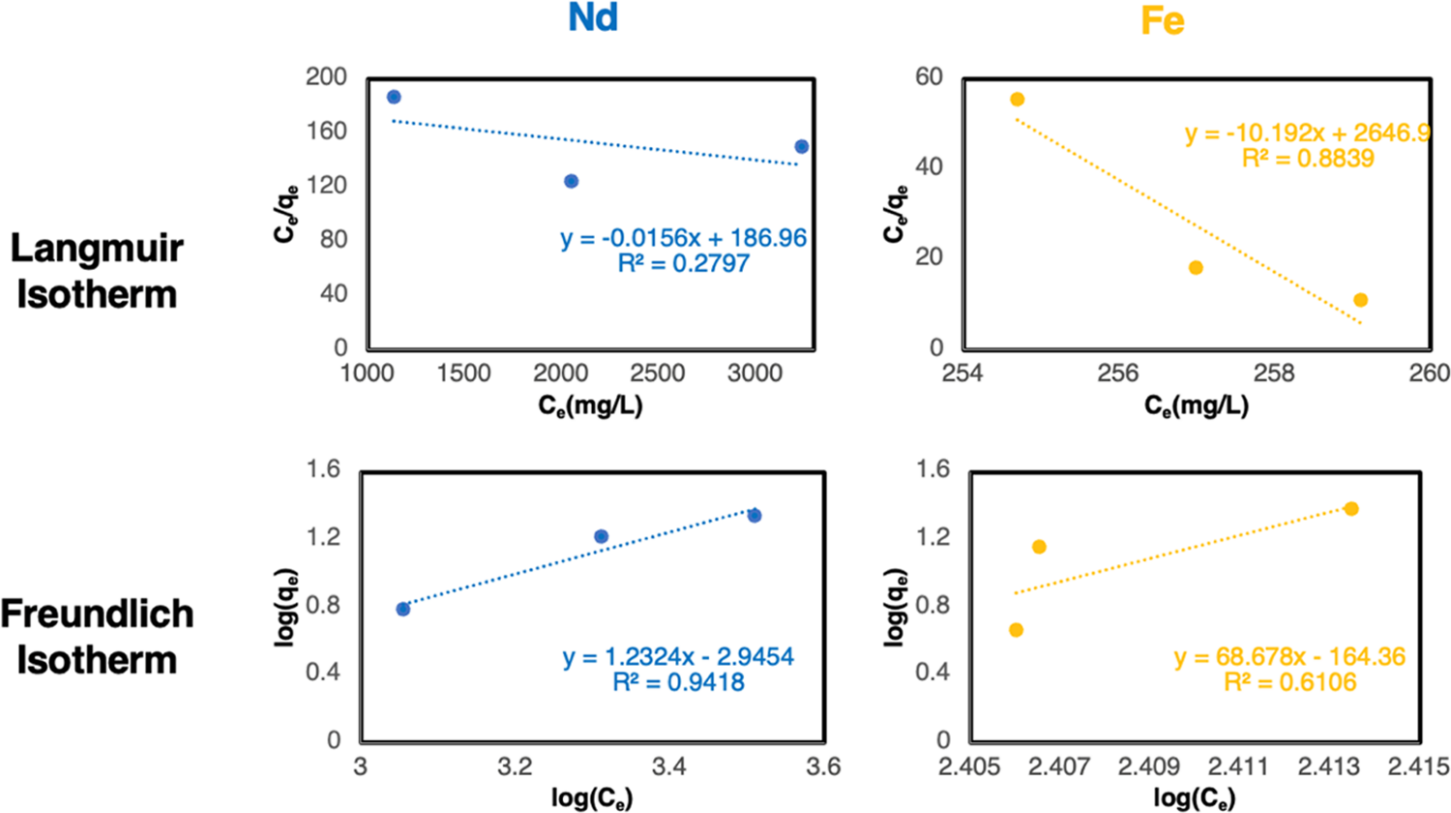
Fitting experimental data to linear Langmuir and linear Freundlich isotherm models for Nd^3+^ and Fe^2+/3+^ ions’ adsorption.

To better understand the adsorption behavior, we investigated the compatibility of the adsorption data of Fe^2+/3+^ and Nd^3+^ ions with the linear Langmuir and Freundlich models. Since REEs exhibit similar behavior, models only on Nd^3+^ ions have been tested. The compatibility of both models with both metal ions is given in Figure 5, and the model constants are given in Table 7. It was understood from the *R*^2^ values that the adsorption of both metal ions was incompatible with the linear version of the Langmuir and Freundlich models. The compatibility of the experimental data with the nonlinear Langmuir and Freundlich models was examined using Microsoft Excel, and the model constants are given in Table 8. It was understood that the adsorption of Nd^3+^ ions was compatible with Freundlich isotherm, and the 1/*n*_f_ value was less than 1, indicating that the reaction was normal adsorption.^22^ Furthermore, it was understood that the adsorption of Fe ions was compatible with both models at high values (see Table 8), but the 1/*n*_f_ value in the nonlinear Freundlich model was found to be greater than 1. For this reason, it was understood that the adsorption process of Fe^2+/3+^ ions was compatible with nonlinear Langmuir, also the *R*_L_ value was less than 1 for 3 different concentration values, and the adsorption reaction was found to be favorable.^22^ While the adsorption of Fe ions occurs in a monolayer and homogeneously, the adsorption of Nd ions occurs in a multilayer and heterogeneously.

**Table 8:** Linear Langmuir/Freundlich and Nonlinear Langmuir/Freundlich Isotherm Models’ Constants

Model	Linear Langmuir			Linear Freundlich		
	*q*_m_ (mg/g)	*K*_L_ (L/mg)	*R*^2^	*n*_f_	*k*_f_ [(mg/g)(L/g)^1/*n*^]	*R*^2^
Nd	–64.1025	–8.34 × 10^–5^	0.2797	0.8114	0.0011	0.9418
Fe	–0.0981	–0.0003	0.8839	0.0145	4.36 × 10^–165^	0.6106

Model	Nonlinear Langmuir			Nonlinear Freundlich		
	*q*_m_ (mg/g)	*K*_L_ (L/mg)	*R*^2^	*n*_f_	*k*_f_ [(mg/g)(L/g)^1/*n*^]	*R*^2^
Nd	189.8722	4.0295 × 10^–5^	0.9968	1.3082	0.0223	0.9987
Fe	236.1737	2.5085 × 10^–5^	0.9999	0.7314	14.2661	0.9999

### 3.4 Characterization of Fe/CaAlg Beads

**Figure 6:**
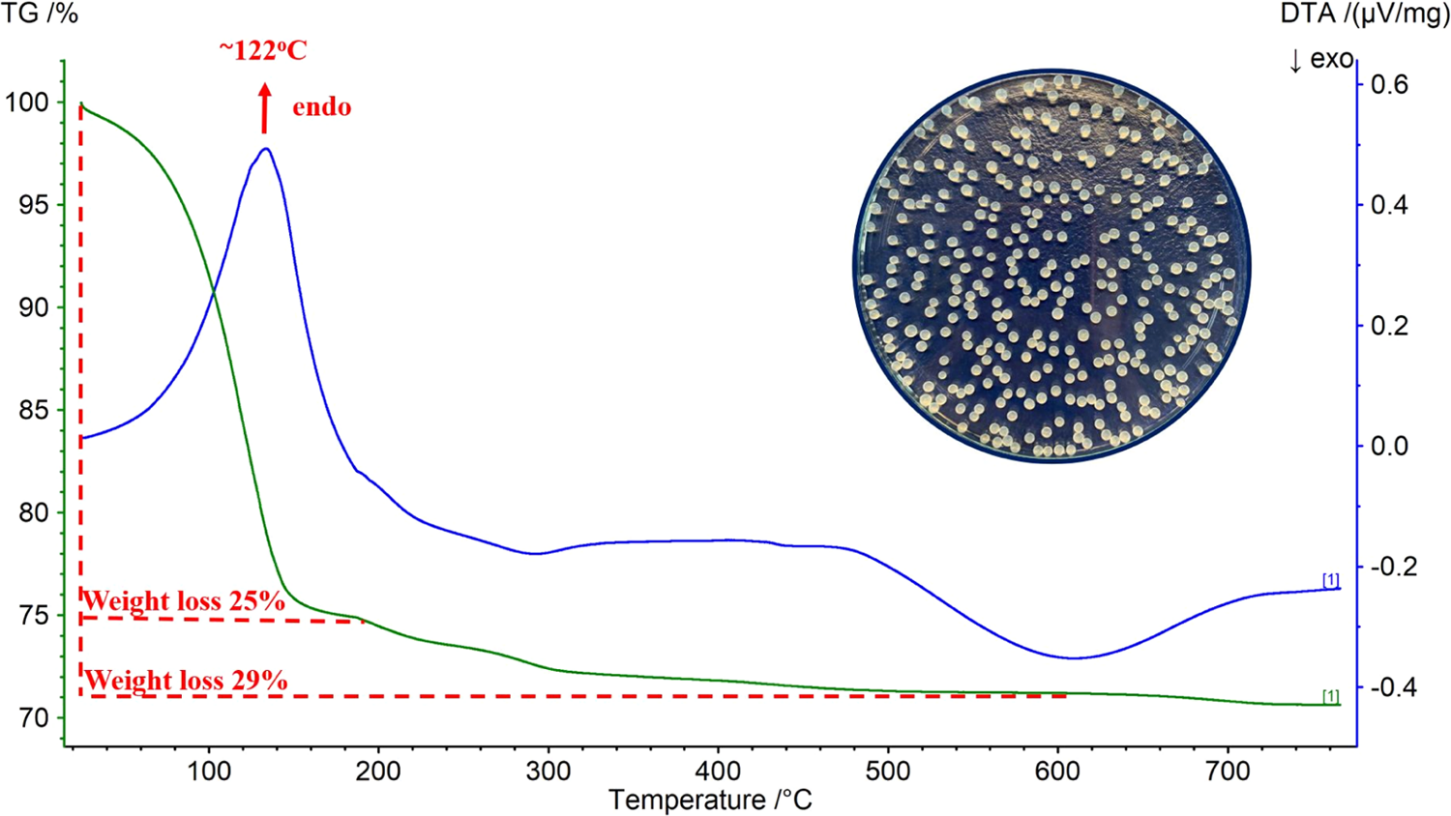
DTA-TG analysis of the Ca alginate beads.

The thermal decomposition behavior of the Ca alginate and Fe alginate beads was studied by DTA-TG analysis. Figure 6 presents the DTA-TG analysis of the CaAlg beads.

**Figure 7:**
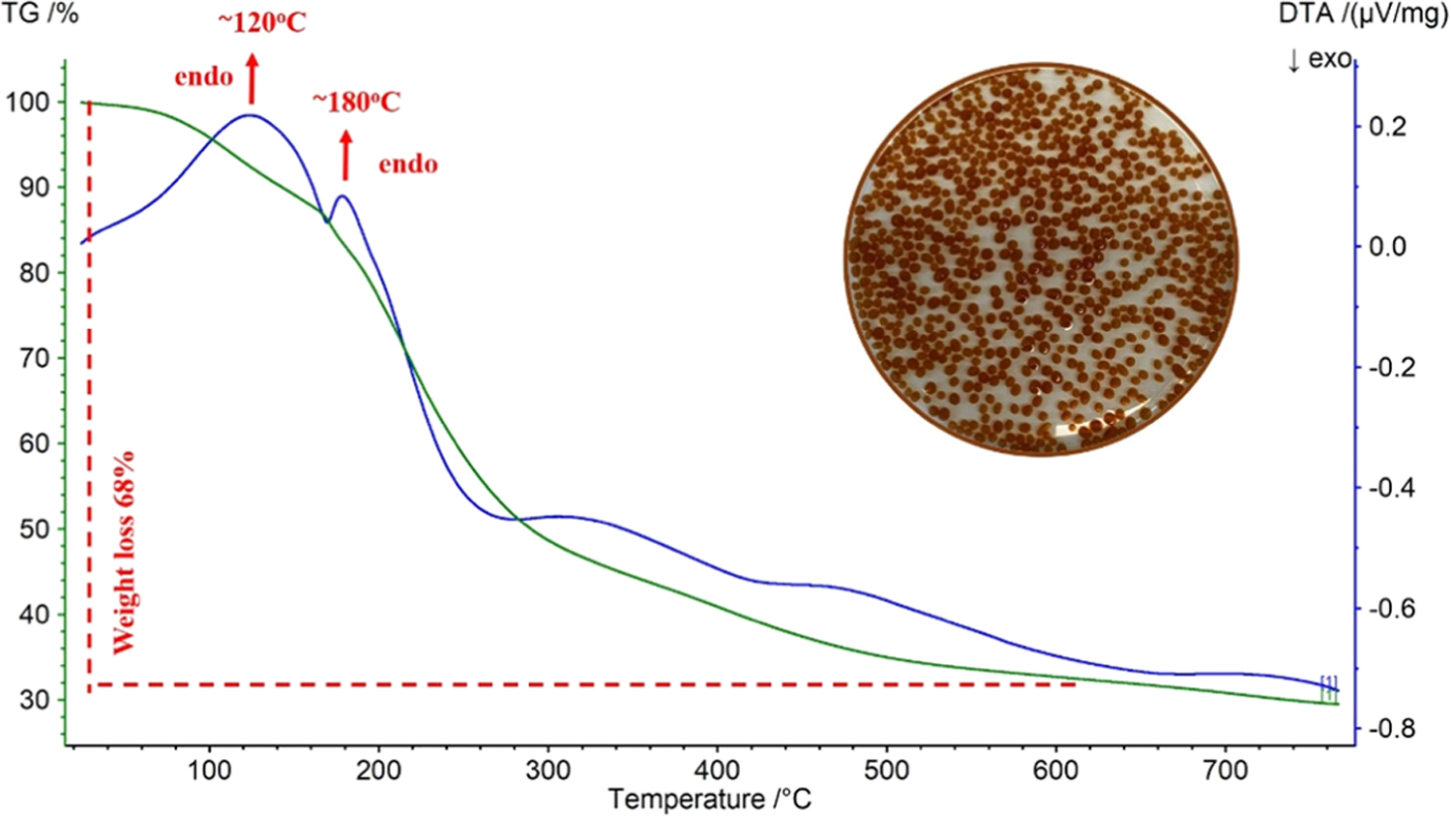
DTA-TG analysis of FeAlg beads.

The DTA curve indicates an endothermic transition at 122 °C. The decomposition process in the range of 25 and 200 °C has a loss of about 25% of the sample mass due to the water loss. The total mass loss of the CaAlg beads is approximately 29%. Figure 7 illustrates the DTA-TG analysis of the FeAlg beads.

The DTA curve shows two endothermic peaks with maxima at 120 and 180 °C. These two peaks on the DTA curve are accompanied by a weight loss of 68%. There appears to be a difference between the thermal decomposition behaviors of CaAlg and FeAlg beads. This can be attributed to the strength of the chelating between the Fe ions and the alginate.

**Figure 8:**
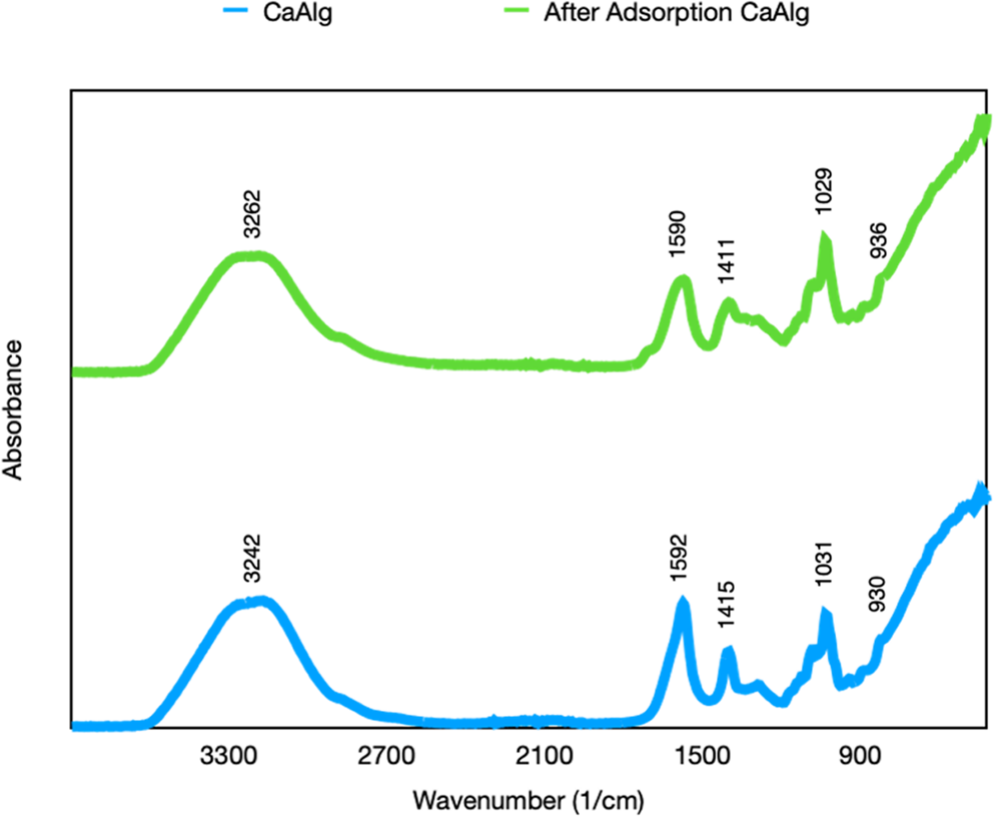
FTIR spectra of CaAlg and FeAlg beads.

The FTIR spectrum of CaAlg and FeAlg gel obtained from the adsorption process is shown in Figure 8. The peak at 3242 cm^–1^ in the CaAlg spectrum refers to the vibration of OH bonds, the peak at 930 cm^–1^ refers to the vibration of the C–O bond, the peaks at 1592 and 1415 cm^–1^ refer to the symmetric and asymmetric carboxyl group vibrations at 3242 cm^–1^, and the peak at 1031 cm^–1^ refers to the pyranose ring.^16,23,31^ The FTIR spectrum of the CaAlg gels obtained as a result of the adsorption process demonstrates that the chemical structure of alginate is preserved. It was observed that the carboxylate peaks at 1592 and 1415 cm^–1^ (shifted to 1590 and 1411 cm^–1^) shifted to the right; furthermore, the pyranose ring bond peak at 1031 cm^–1^ (shifted to 1029 cm^–1^) also shifted to the right. The interaction of ions with different atomic diameters with carboxylate ions causes a shift in these peaks.^16^ During the adsorption process, it was observed through the FTIR results that the Ca^2+^ ions present in the gels were released into the solution, while the metal ions in the solution interacted with the alginate.

## 4 Mass Balance for the Proposed Process

**Figure 9:**
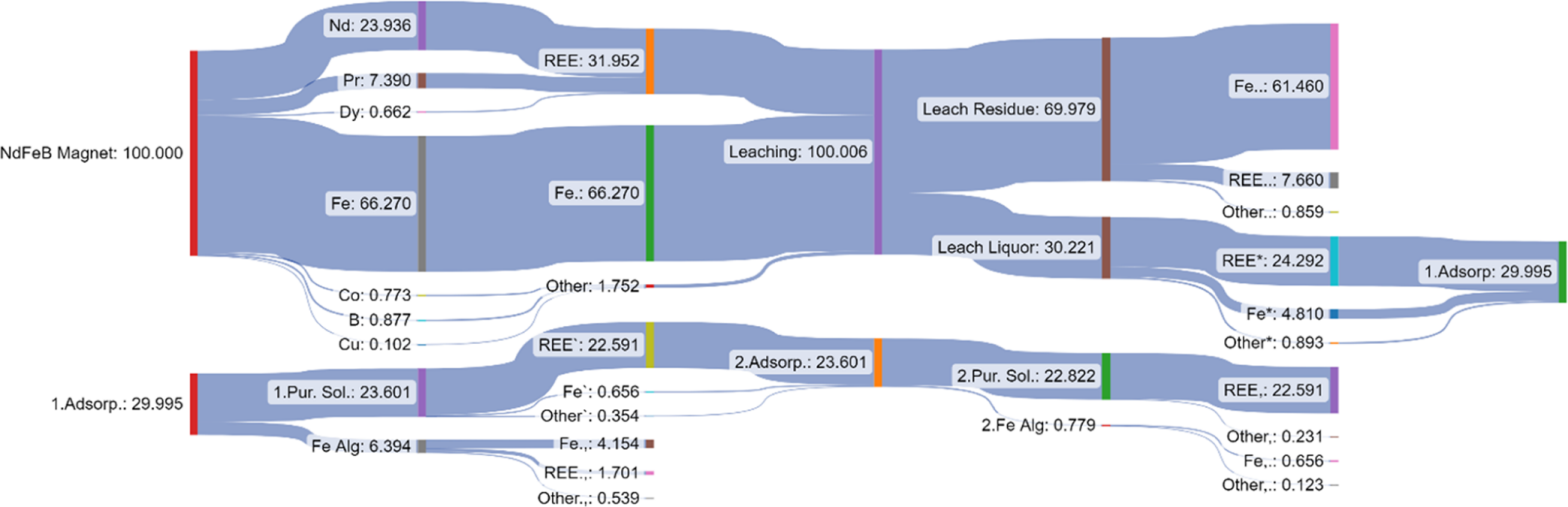
Material flow analysis of the proposed process.

A material flow analysis (MFA) for the recycling of NdFeB magnets by leaching and adsorption was performed under the optimum experimental conditions. All flows are in percentage, and Figure 9 illustrates the Sankey diagram for mass balance of the proposed process with optimum adsorption conditions.

The Sankey diagram presents the overall weight flows obtained from the chemical analysis. The REE flow starts on the left side of the diagram with 31.952%, and 24% of REEs lost during leaching. After iron adsorption under the specified conditions, more than 72% of REEs is recovered. The iron removal and REE recovery efficiencies at leaching, adsorption, and total were calculated and are tabulated in Table 9.

**Table 9:** REE Recovery and Iron Removal Efficiencies

		REE recovery efficiency (%)		
NdFeB magnet powders	leaching	first adsorption	second adsorption	total
	∼77	∼93	∼99	∼72

		Fe removal efficiency (%)		
NdFeB magnet powders	leaching	first adsorption	second adsorption	total
	∼93	∼ 86	∼100	100

Iron removal efficiencies are 93, 86, and 100% in the leaching, first adsorption, and second adsorption steps, respectively. REE recovery efficiencies are 77, 93, and 100% in the leaching, first adsorption, and second adsorption steps, respectively. REEs with a purity of approximately 99% were achieved after the second adsorption process.

## 5 Evaluation of the Antimicrobial Activity of Fe Alginate Beads

**Figure 10:**
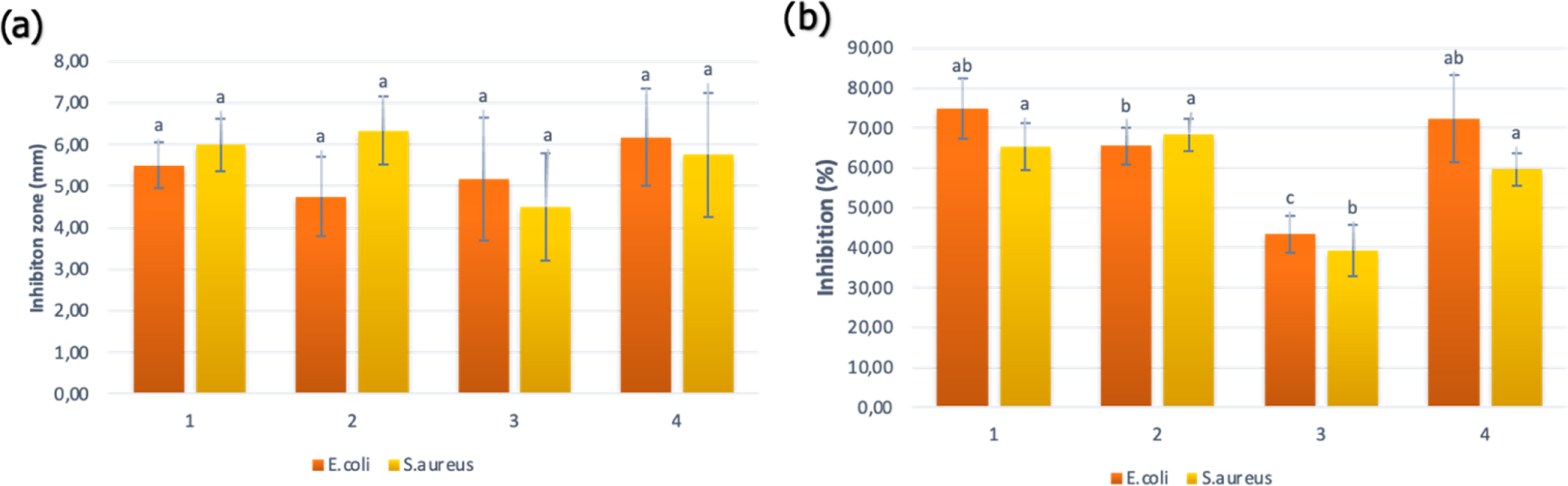
Antimicrobial properties of the material against *E. coli* and S. *aureus*. Zone of inhibition (a) and (%) inhibition (b).

The antibacterial activity of the material has been investigated with disc diffusion and broth dilution methods against Gram-positive *S. aureus* and Gram-negative *E. coli*. Figure 10 shows the results obtained from the conducted analysis.

**Figure 11:**
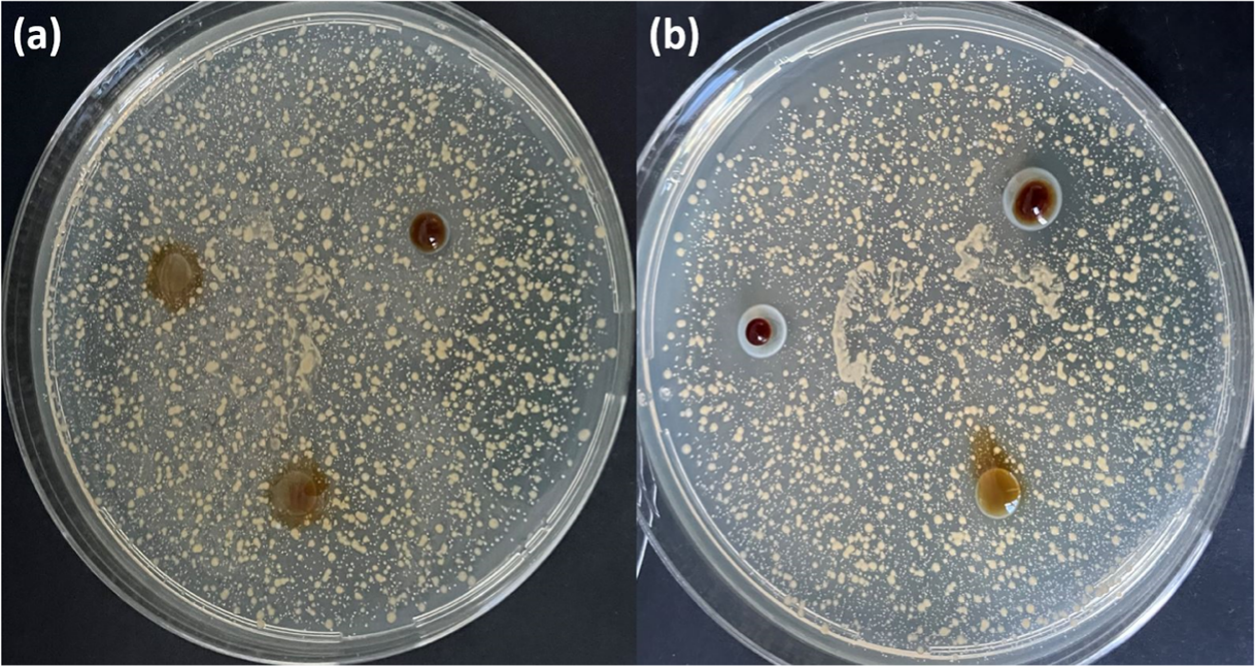
Representative image of antibacterial activity of A2 samples against *S. aureus* (a) and *E. coli* (b).

There were no significant differences between the materials according to the disc diffusion results. An illustration of the antibacterial activity of the A2 samples can be seen in Figure 11. However, the highest inhibition zone among the samples 6.2 ± 1.1 and 5.75 ± 1.5 mm was found against *E. coli* and *S.aureus*. According to the disc diffusion results, there were no significant differences between the materials. This might be explained depending on their release profile on the solid media. Similar impacts have been observed against Gram-positive and Gram-negative bacteria.

As can be seen from Figure 11b, sample 4 was the most effective antibacterial material against *E. coli* and *S. aureus* with inhibitions of 72.37 and 59.60%, respectively. The observed differences in the inhibition of microorganisms can be attributed to differences in bacterial cell wall thickness and cellular structure. Because of its thicker cell walls than those of *E. coli*, *S. aureus* exhibits a greater resistance to bacterial inhibition. Similar results have been reported in the literature.^32^ The increase in antibacterial activity of the material could be explained by the adsorption of a higher iron metal. Meanwhile, the least effective one, sample 3, showed lower antibacterial activity against both *E. coli* and *S. aureus* (*p* < 0.05). In a recent study conducted by Dong et al. (2023), developed tannic acid-assisted fabrication of sodium alginate-based gel beads showed 83.8 and 99.5% inhibition against *S. aureus* and *E. coli*, which is slightly higher than our products.^100^ Hou et al. (2023) reported that AgNP-containing microgel beads showed 7–10 mm inhibition zones via the well diffusion method against *Bacillus subtilis*, *E. coli*, and *S. aureus*.^101^ In addition, Ag and Fe_3_O_4_ nanoparticles in hydrogel beads showed strong antibacterial activity with inhibition zones of 15 and 17 mm, higher than our samples, which might be explained by the prevalence of Ag nanoparticles.^33^ For example, Xu et al. studies showed that the CaAlg membrane did not show antibacterial activity; however, with different amounts of AgNPs, the antibacterial activity of the membrane increased.^34^ Moreover, sodium alginate/chitosan-based hydrogels loaded with metronidazole, an antibiotic, had a higher antibacterial activity with 17–18 mm inhibition zones.^35^

Reactive oxygen species (ROS) are a major source of bactericidal activity in most metal oxide nanoparticles. Their efficiency results from metal ion release and chemical structure.^36^ It is known that the antibacterial activity of the iron oxide nanoparticles depends on the concentration of the nanoparticles.^37^ The process involves reactive oxygen species (ROS), including superoxide radicals, hydroxyl radicals, hydrogen peroxide, and singlet oxygen through the Fenton reaction.^37^ The antibacterial activity may be caused by the interaction of ROS species, proteins, or chemical compounds with iron.^38^ Additionally, in the literature, the antibacterial activity of iron against Gram-positive and Gram-negative bacteria has been reported.^39,40^ Further, there were no significant differences in the inhibition of *S. aureus* among the samples, except sample 3 (*p* < 0.05). These differences might be explained by the lower concentration of adsorption.

## 6 Pilot-Scale Experimental Setup for Fe Alginate Production

Iron removal from the leaching solution via absorption for recovery of REEs was not reported in the literature. The removed iron from NdFeB is a problematic issue since the removed iron cannot be proposed for usage in industry since these irons include various types of impurities; therefore, iron should be treated to find use in industry. In this study, iron is evaluated as an antibacterial material.

**Figure 12:**
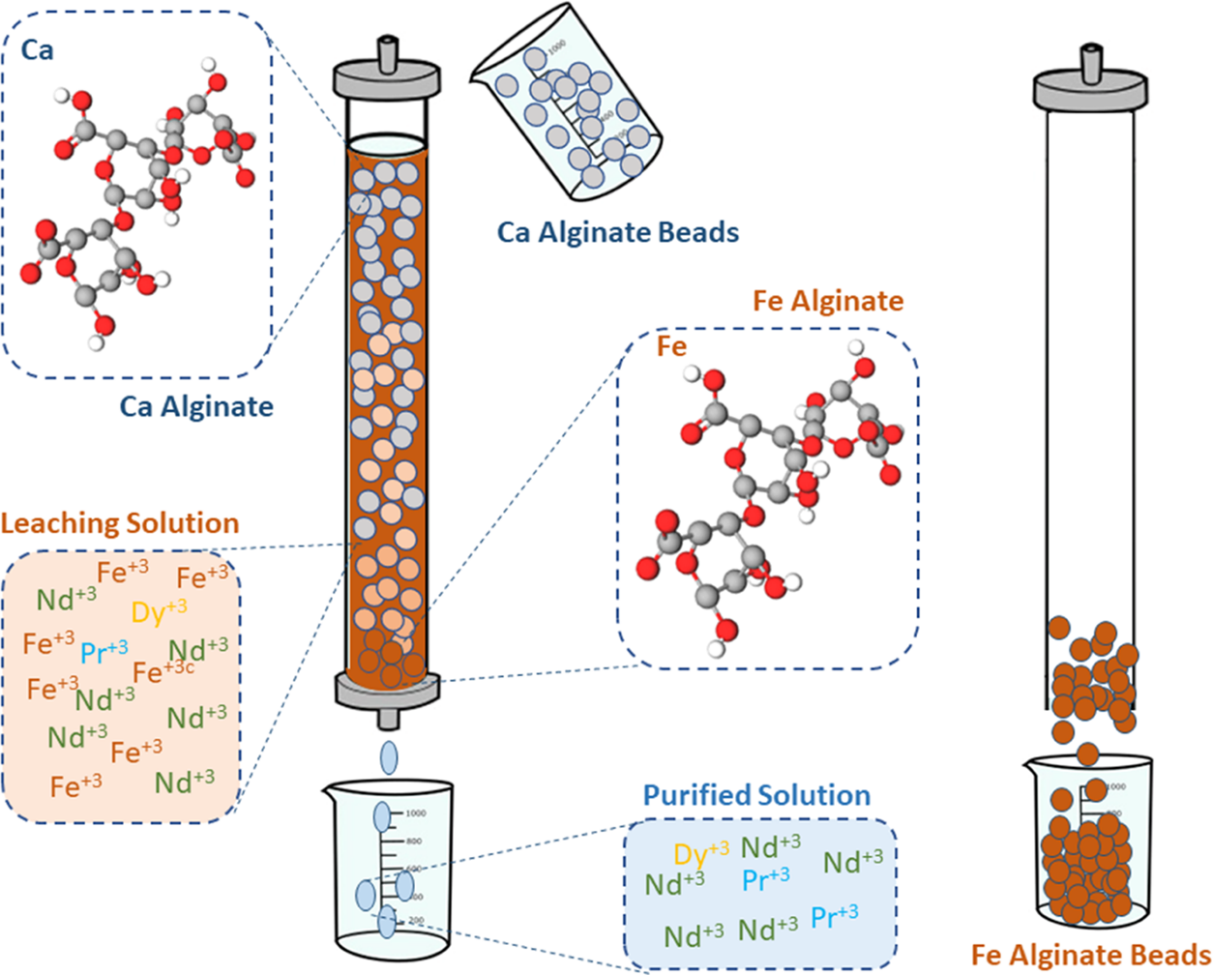
Experimental setup for pilot-scale production of FeAlg.

For pilot-scale production of antibacterial FeAlg and recovery of high-purity REEs, an experimental setup was proposed, as shown in Figure 12.

An adsorption column might be employed to upscale the proposed process for the production of FeAlg from the waste NdFeB magnet. In the adsorption column, Fe can be adsorbed by the fabricated Ca alginate beads. Afterward, the purified solution can be collected in the bottom of the column followed by FeAlg beads being evacuated from the column.

Various types of waste NdFeB magnets are available on the market. The chemical composition of NdFeB magnets is typically as follows: Nd is approximately 29–32 wt %, Fe is approximately 63–68 wt %, and B is approximately 1–1.2%. Those differences do not affect the efficiency of leaching and adsorption process; almost the same results would be obtained in the case of the use of different types of NdFeB magnet waste. The proposed process can be employed for all types of NdFeB magnets on the market.

## Conclusions

The conception of the near**-**zero**-**waste valorization process of waste materials is one of the most interesting matters. In this work, a novel concept for the recycling of REEs from NdFeB and the production of antimicrobial FeAlg was developed. First, the magnet powders were leached with HNO_3_ for selective extraction of REEs. Afterward, the solid and liquid separation was carried out by vacuum setup. There has been still some iron in the leach liquor. CaAlg beads were fabricated by cross-linking sodium alginate in a mixed CaCl_2_ solution. The remaining iron in the leaching solution was adsorbed by the fabricated CaAlg beads. CaAlg beads were shown to be highly effective in adsorbing iron from the leach liquor and their potential for application in the recovery of REEs from NdFeB magnets. The thermal decomposition behavior of the CaAlg and FeAlg beads was investigated by DTA-TG analysis. The difference between the thermal decomposition behaviors of CaAlg and FeAlg beads was observed due to the strength of the chelating between the Fe ions and the alginate. The chemical bonding of the fabricated hydrogels was examined by FTIR analysis and the FTIR spectrum reveals that the cross-linking was derived from Fe^3+^ ions. Moreover, the fabricated FeAlg demonstrated potent antimicrobial activity against *E. coli* and S. *aureus* with inhibitions of 87.21 and 56.25%, respectively. The pilot-scale setup for adsorption studies was proposed to recover high-purity REEs and produce antibacterial FeAlg beads. The suggested process can be employed for NdFeB magnets of all varieties available in the market.

## Notes

E.E.-K. conducted the experiments at IME Process Metallurgy and Metal Recycling, RWTH Aachen University, Aachen, Germany, and is presently employed at the Department of Materials Science and Engineering, Norwegian University of Science and Technology, Trondheim, Norway.
